# Anesthetic management with remimazolam for a pediatric patient with Duchenne muscular dystrophy

**DOI:** 10.1097/MD.0000000000028209

**Published:** 2021-12-10

**Authors:** Yuta Horikoshi, Norifumi Kuratani, Ken Tateno, Hiroshi Hoshijima, Tina Nakamura, Tsutomu Mieda, Katsushi Doi, Hiroshi Nagasaka

**Affiliations:** a Department of Anesthesiology, Saitama Medical University Hospital, Saitama, Japan; b Department of Anesthesia, Saitama Children's Medical Center, Saitama, Japan; c Division of Dento-oral Anesthesiology, Tohoku University Graduate School of Dentistry, Miyagi, Japan.

**Keywords:** anesthesia, duchenne muscular dystrophy, remimazolam

## Abstract

**Rationale:**

With Duchenne muscular dystrophy (DMD) being the most common and most severe type of muscular dystrophy, DMD patients are at risk for complications from general anesthesia due to impaired cardiac and respiratory functions as the pathological condition progresses. In recent years, advances in multidisciplinary treatment have improved the prognosis of DMD patients, and the number of patients requiring surgery has increased. Remimazolam is a benzodiazepine derivative similar to midazolam. Its circulatory stability and the fact that it has an antagonist make it superior to propofol.

There are no reports of pediatric patients with DMD undergoing total intravenous anesthesia with remimazolam.

**Patient concerns:**

**A** 4-year boy was scheduled for single-incision laparoscopic percutaneous extraperitoneal closure for inguinal hernia under general anesthesia, but the surgery was postponed because his serum creatine phosphokinase level was extremely high.

**Diagnosis:**

He was diagnosed with DMD. According to the results of the genetic test, exon deletion of the DMD gene was detected using multiplex ligation-dependent probe amplification, although he had no symptoms of DMD except for elevated serum levels of creatine phosphokinase, etc.

**Intervention:**

He was admitted for the same surgical purpose. Anesthesia was induced with 3 mg of intravenously administered remimazolam. He lost the ability to respond to verbal commands. After the intravenous administration of 100 μg of fentanyl, a continuous infusion of remifentanil (1.0 μg/kg/min) and remimazolam (15 mg/h) was started, and the endotracheal tube was inserted smoothly after the administration of 10 mg of rocuronium with which the muscle twitches disappeared in train-of-four monitoring. At the end of the surgery, 15 mg of flurbiprofen was administered intravenously. After surgery, we injected 40 mg of sugammadex to confirm a train-of-four count of 100%.

**Outcomes:**

Although the dose of remimazolam was reduced to 5 mg/h 30 minutes before the end of the surgery, it took 20 minutes after the discontinuation of remimazolam for the patient to open his eyes upon verbal command. On postoperative Day 2, he was discharged from the hospital without any complications.

**Lessons:**

Remimazolam was shown to be safe to use for general anesthesia in a pediatric patient with DMD.

## 1 Introduction

Duchenne muscular dystrophy (DMD) is characterized as a progressive multisystem neuromuscular disorder. With DMD being the most common and most severe type of muscular dystrophy,^[1]^ DMD patients are at risk for complications under general anesthesia due to impaired cardiac and respiratory functions as the pathological condition progresses.^[2]^ In recent years, advances in multidisciplinary treatment have improved the prognosis of DMD patients,^[2]^ and the number of patients requiring surgery has increased.^[3]^

Remimazolam is a benzodiazepine derivative similar to midazolam and is an intravenous anesthetic with excellent adjustability due to ultrashort-acting pharmacological characteristics for intravenous use. Its circulatory stability and the fact that it has an antagonist (flumazenil) make it superior to propofol.^[4]^

To our knowledge, there are no reports of pediatric patients with DMD undergoing total intravenous anesthesia (TIVA) with remimazolam.

## 2 Case report

Four months ago, a 4-year-old boy (16 kg, 96 cm) was scheduled for single-incision laparoscopic percutaneous extraperitoneal closure for inguinal hernia and umbilical plasty under general anesthesia, but the surgery was postponed because he had an elevated (creatine phosphokinase) level of 41018 U/L (normal range, 59–248). On detailed examination, he was diagnosed with DMD. According to the results of the genetic testing, the deletion of exons 45–52 as the dystrophin-encoding DMD gene deletions were detected using multiplex ligation-dependent probe amplification, although he had no symptoms of DMD except for an elevated creatine phosphokinase level of 53398 U/L (normal range, 59–248), aspartate aminotransferase level of 572 U/L (normal range, 13–30), alanine aminotransferase level of 547 U/L (normal range, 10–42), lactate dehydrogenase level of 2745U/L (normal range, 124–222), and aldolase level of 224.3 U/L (normal range, 2.7–7.5).

Premedication was not used. After arriving at the operating room, oxyhemoglobin saturation measured by pulse oximetry monitoring readings were performed. His oxyhemoglobin saturation measured by pulse oximetry level was 99% under room air. After 3 mg of intravenously administered remimazolam, he lost the ability to respond to verbal commands.

Then, noninvasive blood pressure monitoring, electrocardiogram (ECG), and bispectral index monitoring were performed. After the intravenous administration of 100 μg of fentanyl, a continuous infusion of remifentanil (1.0 μg/kg/min) and remimazolam (15 mg/h) was started, and the endotracheal tube was inserted smoothly after the administration of 10 mg of rocuronium with which the muscle twitches disappeared in train-of-four (TOF) monitoring. The rocuronium dose was determined by TOF monitoring. Although muscle relaxation monitoring was continued to minimize the administration of rocuronium during the surgery, the total use of rocuronium was 10 mg, without additional doses. After the induction of general anesthesia, we maintained the remimazolam dose at 15 mg/h and maintained the remifentanil dose at 1.0 μg/kg/min. Intraoperatively, we determined the doses of remifentanil (1.0 μg/kg/min) and remimazolam (15 mg/h) with reference to our extensive experience with remimazolam in pediatric patients, the signs of his clinical depth, and the relatively high amplitude slow waves in the electroencephalography. However, the bispectral index values showed 70 to 80. We maintained the core body temperature at 37.5°C by using a forced-air warming system and blankets. Twenty-five μg of fentanyl was administered intravenously 20 minutes before the end of the surgery. At the end of the surgery, 15 mg of flurbiprofen axetil was administered intravenously. After the surgery, we injected 40 mg of sugammadex to confirm a TOF count of 100%. Although the dose of remimazolam was reduced to 5 mg/h 30 minutes before the end of the surgery, it took 20 minutes after the discontinuation of remimazolam for the patient to open his eyes upon verbal command. No flumazenil was administered to boost his recovery. A urine myoglobin examination at 1 hour postoperatively showed negative results. On postoperative Day 2, he was discharged from the hospital without any complications.

## 3 Discussion

This case report is the first to present the use of remimazolam under general anesthesia for single-incision laparoscopic percutaneous extraperitoneal closure for inguinal hernia in a pediatric patient with DMD. TIVA is preferred over inhalational anesthetics for patients with DMD.^[5]^ The use of inhalational anesthetics should be avoided since it has been reported that inhalational anesthetics increase the risk of rhabdomyolysis, which can result in hyperkalemia and cardiac arrest.^[5]^ There are some reports that TIVA with remifentanil has been safely performed for DMD patients compared to inhalation anesthesia.^[6,7]^ However, even propofol has been reported to cause rhabdomyolysis.^[8]^

Because remimazolam does not produce injection site pain, which is common in propofol use,^[4]^ and there are reports that general anesthesia with remimazolam has been safely performed in other high-risk patients,^[9]^ we chose to use remimazolam for pediatric anesthesia. Although there are reports of using midazolam, a benzodiazepine receptor agonist similar to remimazolam, as a premedication,^[10,11]^ there are still no reports of remimazolam use for general anesthesia in DMD patients. As a benzodiazepine receptor agonist, remimazolam is an ultrashort-acting sedative/anesthetic and shows high affinity for γ-aminobutyric acid receptors.^[4,12]^ The drug includes the safety profile of benzodiazepines regarding hemodynamic stability with fast onset and offset characteristics and improved controllability.^[4]^

Because there are cases reported in which even apparently healthy children, similar to our present case, suffer from hyperkalemia, resulting in cardiac arrest,^[13]^ careful monitoring, such as ECG monitoring, was required perioperatively in the present case. It is important to recognize that ECG abnormalities are present in infants and young children, as well as in older boys, and precede the development of functional evidence of systolic dysfunction or left ventricular enlargement.^[2,14]^ Fortunately, in the present case, echocardiography and ECG results were within the normal range.

Although forced vital capacity, the pulmonary function parameter is most frequently reported to have predictive value in assessing the risk of respiratory complications for patients with DMD,^[2]^ we could not obtain pulmonary function test data for our patient, despite trying to test his pulmonary function.

While depolarizing muscle relaxants, such as succinylcholine, are widely recognized to be contraindicated in patients with DMD,^[13]^ little information is available on the unfavorable response to nondepolarizing muscle relaxants, but there have been reports of delayed recovery from muscle relaxants.^[6,7]^ In the present case, we used rocuronium, a nondepolarizing muscle relaxant and monitored the patient with a muscle relaxation monitor, but it did not cause any clinical problems.

In this clinical case, stable anesthesia management in a pediatric patient was possible, and postoperative complications, such as impaired respiration and heart functions, were not encountered using remimazolam.

In conclusion, remimazolam was shown to be safe to use for general anesthesia in a pediatric patient with DMD.

## Author contributions

All authors have read and approved the final manuscript.

YH: Writing – original draft and collected the data

NK: Anesthetized the patient and critically revised the article for important intellectual content.

TK: Collected the data and critically revised the article for important intellectual content.

HH: Critically revised the article for important intellectual content.

TN: Collected the data

TM: Anesthetized the patient.

KD: Critically revised the article for important intellectual content.

HN: Collected the data and critically revised the article for important intellectual content.

**Data curation:** Ken Tateno.

**Investigation:** Tina Nakamura.

**Supervision:** Katsushi Doi.

**Writing – original draft:** Yuta Horikoshi.

**Writing – review & editing:** Hiroshi Nagasaka, Norifumi Kuratani, Hiroshi Hoshijima, Tsutomu Mieda.
